# Combined Orthoplastic Approach in Fracture-Related Infections of the Distal Tibia

**DOI:** 10.3390/microorganisms10081640

**Published:** 2022-08-12

**Authors:** Andrea Sambri, Marco Pignatti, Sara Tedeschi, Maria Elisa Lozano Miralles, Claudio Giannini, Michele Fiore, Matteo Filippini, Riccardo Cipriani, Pierluigi Viale, Massimiliano De Paolis

**Affiliations:** 1 Orthopaedics Unit, IRCCS AOU di Bologna, 40138 Bologna, Italy; 2 Plastic Surgery Unit, IRCCS AOU di Bologna, 40138 Bologna, Italy; 3 Department of Medical and Surgical Sciences, University of Bologna, 40138 Bologna, Italy; 4 Infectious Disease Unit, IRCCS AOU di Bologna, 40138 Bologna, Italy

**Keywords:** orthoplastic, distal tibia, free flap, chronic osteomyelitis, allograft, arthrodesis, perossal

## Abstract

This series reports on the treatment of distal tibia (DT) fracture-related infections (FRI) with a combined orthoplastic approach. Thirteen patients were included. In eight patients with extensive bone involvement and in those with a non-healed fracture, the DT was resected (“staged approach”). In five cases, the DT was preserved (“single-stage approach”). A wide debridement was performed, and the cavity was filled with antibiotic-loaded PerOssal beads. All patients had a soft-tissue defect covered by a free vascularized flap (anterolateral thigh perforator flap in eight cases, latissimus dorsi flap in five). At the final follow-up (mean 25 months, range, 13–37), no infection recurrence was observed. In one patient, the persistence of infection was observed, and the patient underwent a repeated debridement. In two cases, a voluminous hematoma was observed. However, none of these complications impacted the final outcome. The successful treatment of FRI depends on proper debridement and obliteration of dead spaces with a flap. Therefore, when dealing with DT FRI, debridement of infected bone and soft tissues must be as radical as required, with no fear of the need for massive reconstructions.

## 1. Introduction

Fracture-related infection (FRI) can be characterized by low-grade inflammation, the presence of sequestra (necrotic and dead bone), and fistulous tracts [1]. The devascularized nature of the sequestrum may protect bacteria from the host immune response, thus limiting the effectiveness of many antibiotics.

Treatment of FRI can be challenging and involves a multidisciplinary team [2,3,4]. It typically combines surgery and a prolonged course of antibiotics [5,6].

Recurrence rates are relatively high after conservative debridement; for chronic osteomyelitis, some authors suggest approaching treatment as a malignant disease [7,8]. Debridement of non-viable, infected, and fibrous tissue along with osseous structures is required, often resulting in large bone and soft tissue defects [9,10].

Contiguous soft tissues may be severely compromised, thus requiring their removal and subsequent reconstruction by plastic surgeons. Moreover, a soft tissue flap can facilitate the distribution of antibiotics and obliterate dead space following the surgical excision [9] Thus, a combined orthoplastic approach can often help to eradicate chronic infections. Many possibilities for wound coverage have been described after radical debridement [11] Muscle and fasciocutaneous free flaps are usually considered the best to fill defects on infected bone and soft tissues [12] Several factors may influence the choice of free flap for lower extremity reconstruction, including the types and the volume of tissue that are deficient and the wound surface area [13].

Beals et al. [14] reported that chronic infections which involved the distal third of the tibia (DT) had a worse prognosis. They hypothesized that it could be due to the type of microorganisms isolated. Moreover, DT FRI has poor fracture healing because of diminished soft-tissue coverage and reduced vascularity in this district [15,16].

The aim of the present study is to report our experience in the treatment of DT FRI with a combined orthoplastic approach, to analyze the complications and recurrence of infection.

## 2. Materials and Methods

This is an observational, retrospective study. The inclusion criteria were as follows: patients with DT FRI, who underwent a combined orthoplastic treatment at a single institution between January 2019 and April 2021, the clinical data, radiological data, and microbiological data were complete, and patients with at least 12 months of follow-up and patients who provided informed consent.

Diagnosis of FRI was made according to criteria defined in FRI consensus definition [17,18,19]. Osteomyelitis was classified according to May et al. [20] and Cierny et al. [21] Each patient’s clinical status was assessed using the Americal Society of Anesthesiologists (ASA) score and Charlson Comorbidity score (CCI). Smoking habits were also recorded.

Debridement of all infected or necrotic bone and soft tissue was completed, similarly to techniques used for tumor surgery [9]. An anteromedial approach to the DT was performed, according to previous surgeries scars, which were excised. After hardware removal, the fracture was clinically assessed for stability [22]. All infected/devitalized skin, sinus tracts, and soft tissues were excised. Histolopathological assessment was performed to confirm infection.

For patients with broad or circumferential involvement of cortical bone and in those with a non-healed fracture, the DT was resected down to the ankle joint (“staged approach”).

A temporary arthrodesis was performed with a static spacer, made of metal K wires and antibiotic-loaded polymethylmethacrylate [23].

**Figure 1:**
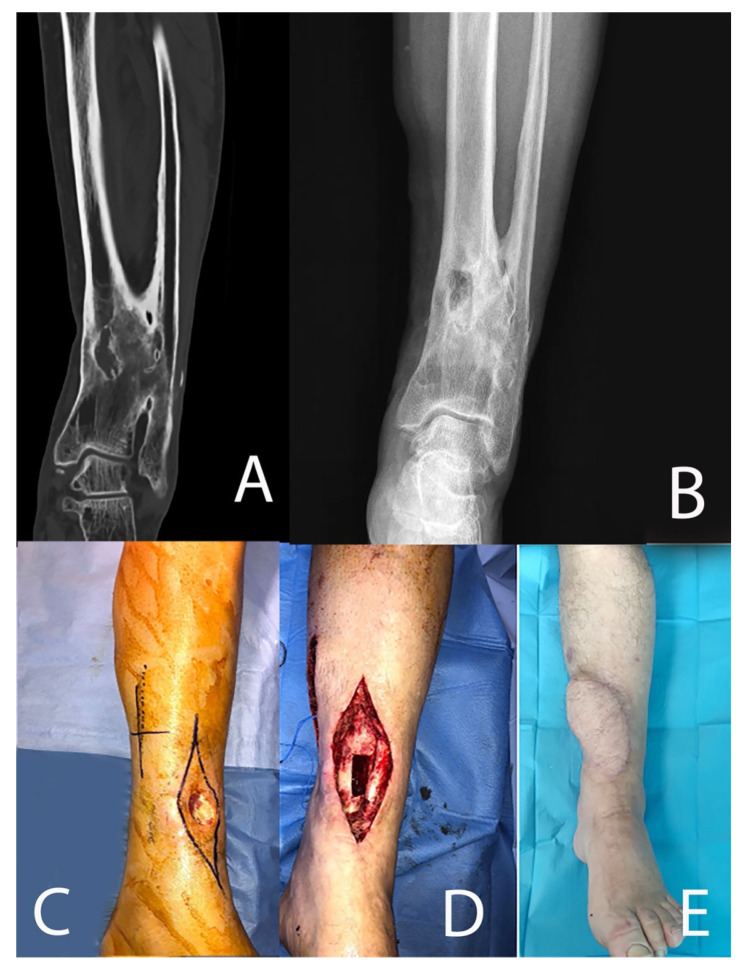
Chronic osteomyelitis of the distal tibia ((**A**): pre-operative coronal view CT scan) treated with curettage and antibiotic-loaded beads ((**B**): anteroposterior X-rays). The discharging sinus (**C**) was excised together with infected soft tissues (**D**) and reconstructed with a free flap ((**E**): 4 months follow-up).

In the case of minor bone defects and healed pseudoarthrosis, the DT was preserved (“single-stage approach”). A wide debridement was performed, and all devitalized bone was removed with curettes and a high-speed burr. The cavity was washed with a hydrogen peroxide solution and dried by gauze packing. It was then filled with PerOssal^®^ (Osartis, Münster, Germany), composed for 51.5% of nanocrystalline hydroxyapatites and for 48.5% of Calcium sulfate [24]. PerOssal^®^ can be loaded with a large choice of antibiotic drugs, based on previous culture samples and epidemiology data [24,25] (Figure 1).

At the time of the first stage, all patients had the skin and soft tissue defect covered by a free vascularized flap (either anterolateral thigh perforator flap—ALT or latissimus dorsi flap) with both artery and vein anastomosis on posterior tibial vessels [6,10,26,27].

Empirical intravenous broad-spectrum antibiotic therapy was begun immediately after surgery after at least five tissue samples were harvested from representative parts and sent for culture. All patients were managed in collaboration with a dedicated infectious diseases specialist. After culture results antibiotic therapy was de-escalated, using an oral regimen whenever possible. Antibiotics were continued for 4–6 weeks until normalization of C reactive protein (CRP).

In the case of a staged approach, after an “antibiotic holiday” of 2 weeks since stopping antibiotic therapy, if persistence of infection was supposed clinically and on lab tests, a repeated debridement with spacer exchange was performed and antibiotics were re-started. In case of no evidence of persistent infection, the patient underwent the reconstructive stage.

**Figure 2:**
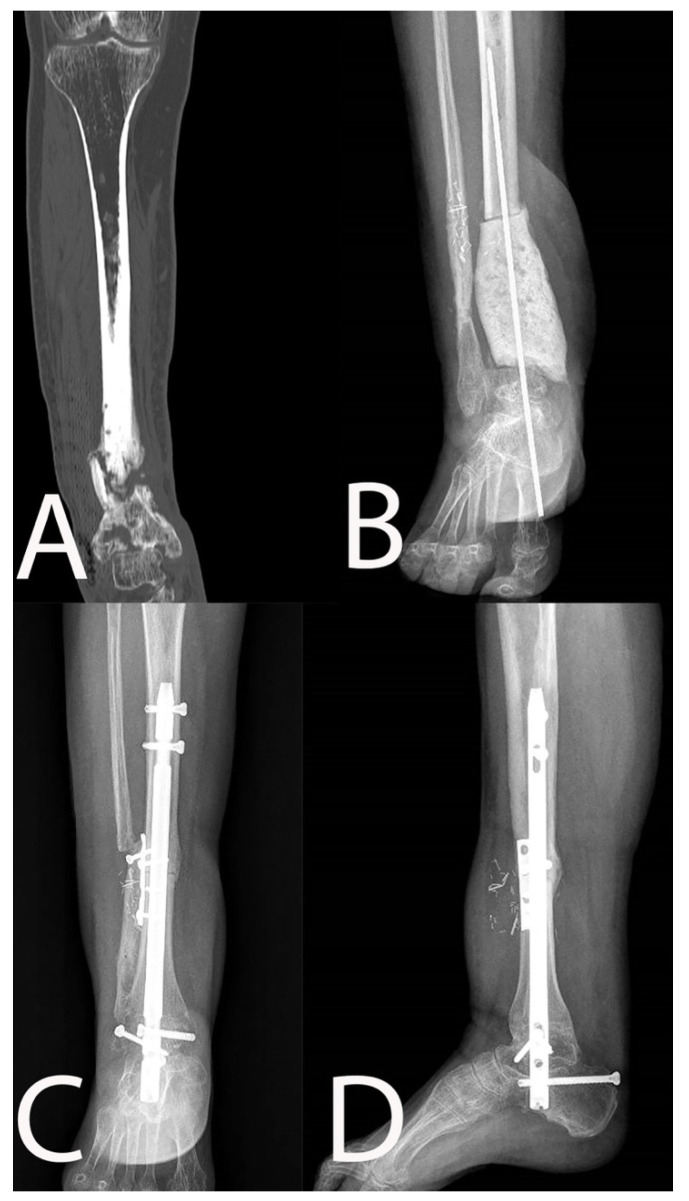
Chronic osteomyelitis of the distal tibia with non-healed pseudoarthrosis ((**A**): pre-operative coronal view CT scan) treated with resection and antibiotic-loaded spacer ((**B**): anteroposterior X-rays). Reconstruction was performed in ankle arthrodesis using a massive bone allograft and a nail (5 months follow-up X-rays, (**C**): anteroposterior view; (**D**): lateral view).

In patients undergoing a staged approach, reconstruction was performed with ankle arthrodesis (combined tibiotalar and subtalar arthrodesis). The foot was fixed in 5 degrees of dorsiflexion and 5 degrees of valgus. In all patients but one, the defect was filled with a massive bone allograft and arthrodesis was fixed with a retrograde nail (Figure 2).

**Figure 3:**
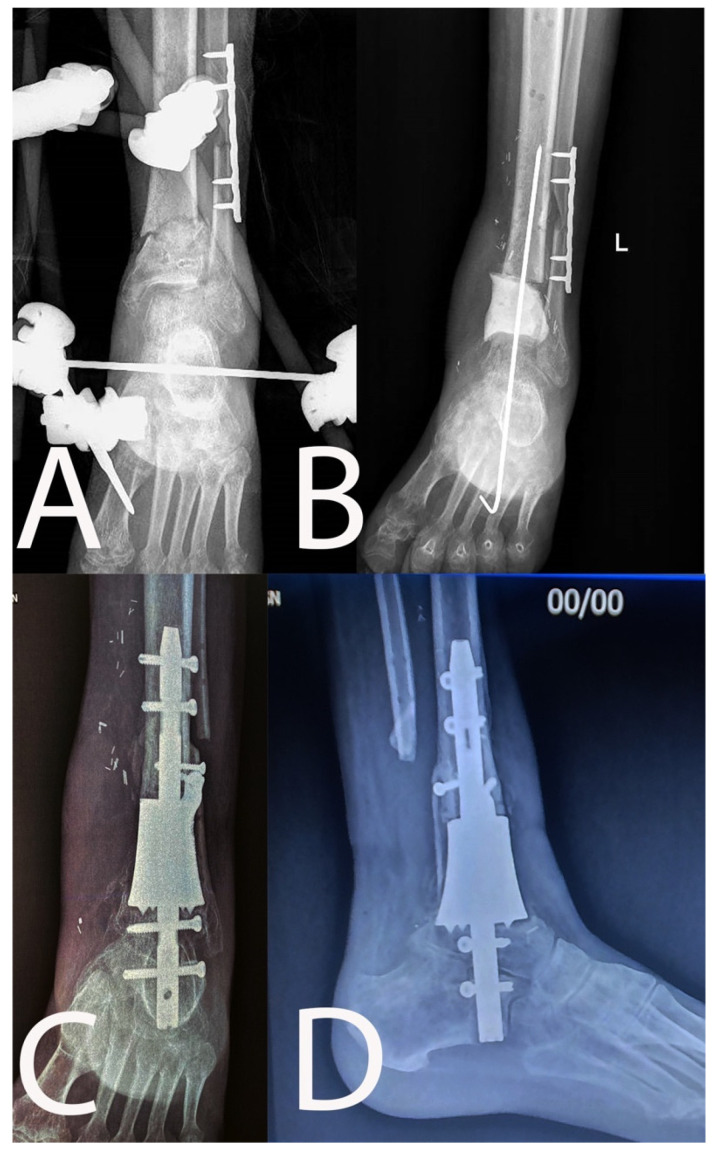
Chronic osteomyelitis of the distal tibia with non-healed pseudoarthrosis ((**A**): pre-operative anteroposterior X-rays) treated with resection and antibiotic-loaded spacer ((**B**): anteroposterior X-rays). Reconstruction was performed in ankle arthrodesis using a silver-coated custom-made prosthesis (7 months follow-up X-rays, (**C**): anteroposterior view, (**D**): lateral view).

The autologous homolateral fibula was transposed and fixed to the graft to improve reconstruction healing. Allografts were taken from the local bone bank and stored according to standard musculoskeletal banking rules. [28] One patient (#4) was reconstructed with a custom-made silver-coated prosthesis (Waldemar Link GmbH & Co. KG, Hamburg, Germany) (Figure 3).

Patients were evaluated with DT X-rays and blood CRP monthly during the first 6 months and every 3 months up to the 2nd year after surgery.

Eradication of infection at a minimum of 12 months follow-up after surgery was the main outcome. Recurrence of infection was defined as sinus formation, additional surgery for FRI, or the need for chronic antibiotic treatment for persistent symptoms. Any wound healing problems, postoperative complications, and need for reoperation were considered secondary outcomes.

## 3. Results

Thirteen patients (three females and ten males) were included (Table 1). The mean age at the time of surgery was 49 years (range, 18–64). All patients had a discharging sinus tract. All patients were preoperatively evaluated with X-rays, magnetic resonance imaging (MRI), and computerized tomography (CT) of the DT [29].

All patients but two had the fracture treated surgically. Four patients had previously undergone hardware removal and surgical debridement elsewhere.

Among the eight patients who underwent a staged approach, the mean resection length was 86 mm (range 60–120 mm) (Table 2).

In four infections, multiple bacteria were isolated from intraoperative tissue samples.

All patients but one required only one spacer, whereas one patient required a repeated debridement with a spacer exchange for persistent infection. The second stage was performed after a mean of 77 days (range, 51–118)

At the final follow-up (mean 25 months, range, 13–37), all patients were alive and had a stable joint at the clinical and radiological examination. All patients who underwent a staged approach had bone union reached after a mean of 6.4 months (range 3–11). No infection recurrence was observed.

In one patient, the persistence of infection was observed, and the patient underwent a repeated debridement 49 days after index surgery. The same patient experienced a massive deep venous thrombosis, thus requiring the placement of a caval filter.

In two cases, a voluminous hematoma developed during the first 48 h after the first surgery at the ankle site, which required surgical evacuation. However, none of these complications impacted the final outcome.

## 4. Discussion

The key to the successful management of FRI is appropriate surgical and antimicrobial treatment; a multidisciplinary approach is needed [3].

A comparison of treatment modalities for FRI is difficult to achieve as most of the series are heterogeneous and include children with adults, multiple bones, various treatments, variability of pathogens, and systemic host factors. Moreover, many studies adopted different definitions to determine success (e.g., a combination of eradication and fracture healing, functional outcome, or absence of amputation). To the best of our knowledge, this is the first series reporting on a selected cohort of patients affected by DT FRI, treated with a combined orthoplastic approach.

No recurrence of infection was observed in our series, thus showing that the vascularized soft-tissue cover provided by free flaps at the time of surgical debridement can help in fighting chronic/late-onset FRI. The use of a free flap, despite increasing surgical difficulties and the incidence of possible early complications, can offer exciting medium to long-term results. This was evident with both treatments (single-stage vs. staged approach). In five cases, a more conservative approach was possible. The use of antibiotic-loaded bone substitutes permits filling bone cavities after surgical debridement. They offer a scaffold for new bone formation in order to restore the mechanical and physiological properties of the bone [30]. Previous reports highlighted that surgical debridement plus antibiotic-loaded bone substitutes lead to a high healing rate compared with surgical debridement alone [25,31,32,33]. In eight patients with extensive or circumferential involvement and in those with non-healed pseudoarthrosis, the DT was sacrificed, thus leading to a massive bone defect. The appropriate method of reconstruction for patients with a large DT defect remains controversial, with excellent results coming from the musculoskeletal oncology field, using a massive homologous graft to perform an ankle arthrodesis [28]. In one case, the massive defect was reconstructed with a custom-made prosthesis fixed in ankle arthrodesis, thanks to the potential additional benefits of silver coating in fighting infections [34,35].

In the whole series, soft tissue reconstruction was performed at the first stage of treatment. Local flaps may not help to cover defects involving DT; microvascular muscle transfer has been proposed to overcome this problem. Previous studies demonstrated that free flaps could increase blood flow and the delivery of antibiotics, enhance phagocytic activity and reduce bacterial counts [36]. Free flaps can also help to expedite bone healing in the early phases of repair [37]. The choice between muscle or fasciocutaneous flaps should be based on the size, the depth, the location of the wound, and the length of the pedicle required [27]. Weiland et al. [38] reported on the use of latissimus dorsi muscle flaps for these defects. However, free fasciocutaneous flaps can give similar functional outcomes as muscle flaps [39,40]. DT requires thin and pliable soft-tissue coverage of exposed bones, tendons, and joints [41]. Advantages of fasciocutaneous flaps include less bulkiness and a better aesthetic outcome [26,27]. Nonetheless, they cannot fill large defects and they need a skin graft at the donor site. As previously reported by other series, we observed that the actual type of flap used for coverage, if well vascularized and implanted after an adequate debridement, is less critical in determining the final outcome [11,26,42]. In the present series, the survival rate of free flaps of 100% is comparable to previously published data by Hong et al. [43].

Some limitations must be acknowledged as it is a retrospective study. Moreover, the sample size is small. However, we report a selected homogeneous cohort of patients affected by DT FRI, treated with a combined orthoplastic approach. Another limitation is the relatively short follow-up, which does not allow for final conclusions.

## 5. Conclusions

Surgical debridement, including massive debridement with bone resection, combined with the use of vascularized free flap, followed by eventual reconstruction of the bone defect, has shown excellent results in terms of infection control in the treatment of DT FRI in this series. With the limitations related to the low number of patients analyzed, this study strengthens the hypothesis that adequate debridement together with obliteration of dead spaces and restoration of local blood supply using an effective flap may represent an option to be primarily contemplated in all complex infections involving the DT with soft tissue impairment.

## Figures and Tables

**Table 1:** Patients’ characteristics at baseline. ASA score: American Society of Anesthesiologists classification; CCI: Charlson Comorbidity Index.

Patient	Age at Surgery and Sex	ASA Score	Smoker	CCI	Pseudoarthrosis	MAY Classification	Cierny–Mader Classification
1	63, M	2	No	4	Yes	Type 4	Type 4 Bl
2	62, M	2	No	2	No	Type 2	Type 3 A
3	50, M	2	Yes	2	Yes	Type 3	Type 4 A
4	45, M	2	Yes	2	Yes	Type 4	Type 4 A
5	57, M	2	No	3	No	Type 2	Type 3 A
6	33, M	1	Yes	3	Yes	Type 4	Type 4 A
7	64, F	3	Yes	5	Yes	Type 3	Type 4 A
8	33, M	1	Yes	2	No	Type 1	Type 3 Bs
9	59, M	2	No	4	Yes	Type 4	Type 4 Bl
10	58, M	3	No	5	Yes	Type 4	Type 4 Bl
11	18, F	1	No	1	No	Type 2	Type 3 A
12	46, F	2	Yes	3	No	Type 1	Type 2 A
13	30, M	2	Yes	3	No	Type 1	Type 3 A

**Table 2:** Treatment and follow-up data. MR: methicillin-resistant; MS: methicillin-sensitive; SA: *Staphylococcus aureus*; *CoNS*: coagulase-negative staphylococcus.

Patient #	Surgery (Resection vs. Debridement)	Hardware Removal	Length of Resection (mm)	Local Antibiotic	Bacteria	Free Flap	Follow-Up (Months)	Recurrence of Infection	Complications
1	Resection	Yes	120		MRSA + *E. faecalis* + *P. stutzeri*	ALT	37	No	
2	Debridement	Yes		Gentamicin	MRSA	ALT	25	No	
3	Resection	No	60		*P. aeruginosa*	Latissimus dorsi	22	No	
4	Resection	Yes	75		*E. coli*	ALT	33	No	Hematoma
5	Debridement	Yes		Rifampicin	*E. faecium* + MRSA	ALT	18	No	
6	Resection	No	63		MS CoNS	ALT	16	No	Persistence of infection, DVT
7	Resection	Yes	60		MR CoNS	ALT	19	No	
8	Debridement	Yes		Rifampicin	MSSA	Latissimus dorsi	27	No	
9	Resection	Yes	100		MRSA + *P. aeruginosa* + *E. faecalis*	ALT	30	No	
10	Resection	No	100		MSSA	ALT	34	No	
11	Resection	No	110		MRSA + *E. coli* + *E. faecium*	Latissimus dorsi	29	No	
12	Debridement	Yes		Gentamicin	MS CoNS	Latissimus dorsi	15	No	
13	Debridement	Yes		Rifampicin	MSSA	Latissimus dorsi	13	No	Hematoma

## Data Availability

The data presented in this study are available on request from the corresponding author.
